# Increased expression of cardiac IL-17 after burn

**DOI:** 10.1186/1476-9255-7-38

**Published:** 2010-07-27

**Authors:** Richard F Oppeltz, Qiong Zhang, Meenakshi Rani, Jennifer R Sasaki, Martin G Schwacha

**Affiliations:** 1 Department of Surgery, The University of Texas Health Science Center, San Antonio, TX, 78229, USA

## Abstract

**Background:**

Cardiac dysfunction is a common complication associated with major burns. While recent findings have linked the Th-17 T-cell response to the development of autoimmune myocarditis, the role of IL-17 and the Th-17 T-cell response in the development of post-burn cardiac dysfunction remains unknown.

**Methods:**

Male C57BL/6 mice were subjected to a major burn (3^rd ^degree, 25% TBSA) or sham treatment. Three hours after injury plasma and tissue (i.e., heart, lung, liver, small intestine) samples were collected and analyzed for the expression of Th-17 cytokine (*i.e*., IL-6, IL-17, IL-22, IL-23, TGF-β) levels by ELISA.

**Results:**

Cardiac tissue levels of the Th-17 cytokines, IL-6, IL-17 and IL-22 were significantly elevated at 3 hrs after burn as compared to sham levels. IL-17 was analyzed 1, 3 and 7 days after burn and showed a return to baseline levels and without a difference in the burn group. Burn-induced alterations in the level of these cytokines in plasma or other tissues were not evident. The cardiac Th-17 cytokine response after burn injury was specific, as cardiac levels of Th-1 (IFN-γ) and Th-2 (IL-10) cytokines were not significantly affected after injury. The cardiac Th-17 response correlated with a significant increase in Troponin levels at 3 hr. after burn.

**Conclusion:**

These findings indicate that early after burn, cardiac tissue is associated with significantly elevated levels of Th-17 cytokines. The early Th-17 response after burn appears to be specific for cardiac tissue and may promote myocardial inflammation and dysfunction associated with this form of trauma.

## Background

Burn injury initiates changes in the immune function that result in local and systemic inflammation that can lead to life-threatening end-organ dysfunction. Cardiac dysfunction after burn has been well described and has been shown to play an important role in patient outcome [1]. Several studies have been conducted to explain the pathways involved in myocardial dysfunction after burn since the early studies of Blalock in the 1930's [2]. Excessive production of pro-inflammatory cytokines, interleukin (IL)-6 IL-1β and tumor necrosis factor-α (TNF-α) have been associated with the cardiac cell damage [3]. Studies have shown differences in the cytokine expression profile in severely burned patients and in the magnitude of the systemic and compartmental inflammatory response correlated with progressive left ventricular contraction and relaxation defects, achieving a nadir with 40% of TBSA burn [3,4]. While studies have implicated cardiomyocytes as a cellular source of pro-inflammatory cytokines after burn, other cell populations are also clearly important [5,6].

Recently a novel class of T-helper cells, called Th-17 cells, has been found to secrete the pro-inflammatory cytokine IL-17 [7]. This recently discovered cytokine appears to play an important role in inflammation and autoimmunity. Moreover, in addition to IL-17, other cytokines such as IL-6 and TGF-β, normally associated with inflammation, are also associated with Th-17 cells. In this regard, the Th-17 response has been recently implicated in the response to several models of infection [8]. This suggests that the Th-17 response may be important in the inflammatory response after burn.

The importance of cardiomyocyte injury after burn has been emphasized in recent years, however, its pathogenesis, has not been fully clarified. In the current study we examined the role of the Th-17 mediated inflammatory response in the development of the cardiac injury after burn in a mouse model.

## Methods

### Animals

C57BL/6 male mice (18 to 22 g; 8 to 10 wk of age, Charles River Laboratories, Wilmington, MA) were used for all experiments. The mice were allowed to acclimatize in the animal facility for at least 1 week prior to experimentation. Animals were randomly assigned into either a thermal injury group or a sham treatment group. The experiments in this study were approved by the Institutional Animal Care and Use Committee of the University of Texas Health Science Center at San Antonio, and were performed in accordance with the National Institutes of Health guidelines for the care and handling of laboratory animals.

### Thermal injury procedure

Mice received a scald burn as described previously [9]. Briefly, the mice were anesthetized by intraperitoneal (IP) injection of ketamine/xylazine, and the dorsal surface was shaved. The anesthetized mouse was placed in a custom insulated mold exposing 12.5% of their total body surface area (TBSA) along the right dorsum. The mold was immersed in 70°C water for 10 sec to produce a 3rd degree burn. The burn procedure was repeated on the left dorsum yielding a total burn size of 25% TBSA. The mice were then resuscitated with 1 ml of Ringer's lactate solution administered by intraperitoneal injection and returned to their cages. The cages were placed on a heating pad for 2 hr until the mice were fully awake, at which time they were returned to the animal facility. Sham treatment consisted of anesthesia and resuscitation with Ringer's lactate solution only.

### Tissue collection and processing

At 3 hr after injury the mice were euthanized and tissue samples collected (plasma, heart, lung, liver, and small intestine). The tissue samples were snap frozen in liquid nitrogen and stored at -80°C prior to analysis.

### Cytokine, Troponin-I, Myleoperoxidase and HMGB-1 determinations

Tissue samples were homogenized in protease inhibitor cocktail as previously described prior to analysis [10]. Plasma samples were not treated. Tissue levels of Th-17 (IL-6, IL-17, IL-22, IL-23, TGF-β) Th-1 (IFN-γ), and Th-2 (IL-10) cytokines were determined by ELISA according to the manufacturer's recommendations(R&D Systems) Plasma cardiac specific Troponin-I, HMGB-1 levels and cardiac myleoperoxidase (MPO) levels were determined by ELISA similarly, (Genway, Shino-Test Corp, Hycult biotechnology). Values obtained were normalized to total protein of the tissue homogenate as determined by BCA assay.

### Statistical analysis

Data are expressed as mean ± SE. Comparisons were analyzed using ANOVA. A *P *value of < 0.05 was considered to be statistically significant for all analyses.

## Results

### Burn induces an increased Th-17 response in the heart at 3 hours after injury

**Figure 1:**
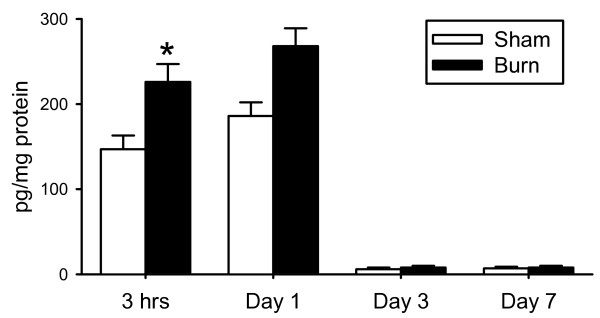
***Cardiac IL-17*: Cardiac tissue at 3 hrs, 1 day, 3 days and 7 days was assessed for IL-17 content as described in the Materials and methods**. Data are mean ± SE for 5-6 mice/group. *P < 0.05 as compared to sham.

Sham and burn groups were composed of 6 and 5 animals, respectively. There were no animal deaths after burn or sham procedures. At 3 hrs, 1 day, 3 days and 7 days after the burn or sham treatment, animals were euthanized and the heart removed. As shown in figure 1, burn caused a significant (*P *< 0.05) elevation in the cardiac levels of IL-17, 3 hours after injury as compared to the sham animals. One day after burn, IL-17 remained elevated, however, without significant difference from the respective sham group. Sham levels of IL-17 were significantly (P < 0.05) greater at 3 hrs and 1 day as compared with days 3 and 7. This early cytokine response is likely related to volume loading early after resuscitation, as both sham and burn mice received resuscitation fluid. Based on the observation that the IL-17 response was significantly higher at 3 hrs after burn, all subsequent analysis was conducted on that group of animals.

**Figure 2:**
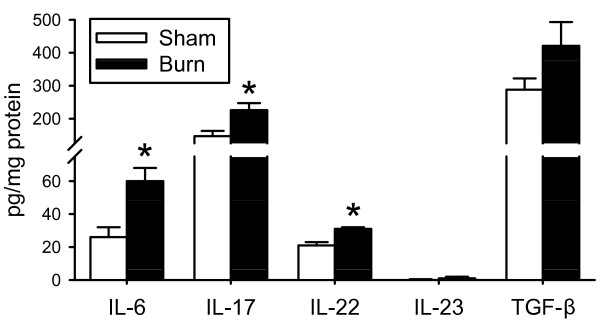
***Cardiac Th-17 Cytokines*: Cardiac tissue at 3 hrs after burn or sham procedure was assessed for content of Th-17 cytokines as described in the Materials and Methods**. Data are mean ± SE for 5-6 mice/group. Data shown for IL-17 is the same as that shown for IL-17 at 3 hrs in Fig.1 and is included for comparative purposes. *P < 0.05 as compared to sham.

In addition to IL-17, we analyzed the heart for the Th-17 family cytokines IL-6, IL-22, IL-23 and TGF-β. As shown in figure 2, burn induced an early myocardial inflammation as evidenced by significantly higher (*P *< 0.05) levels of IL-17, IL-6 and IL-22 in the burn group as compared to the sham animals. The TGF-β response was not different between groups and IL-23 was virtually undetectable in cardiac tissue samples.

We also investigated the effects of burn in other tissues at 3 hrs after injury. Table 1 summarizes the findings for plasma, liver, lung and small intestine. In contrast to the cardiac response, no significant differences were found in those tissues with regard to IL-6, IL-17 and IL-22 levels.

**Table 1:** Tissue levels of IL-6, IL-17 and IL-22.

		Plasma	Lung	Liver	**S.I**.
IL-17	Sham	n/d	175 ± 60	421 ± 96	19 ± 13
	Burn	n/d	133 ± 60	309 ± 63	18 ± 14

IL-6	Sham	n/d	72 ± 20	103 ± 15	4 ± 3
	Burn	n/d	53 ± 8	70 ± 15	4 ± 3

IL-22	Sham	n/d	50 ± 17	66 ± 5	6 ± 2
	Burn	n/d	20 ± 3	59 ± 7	5 ± 2

Plasma, lung, liver and small intestine levels of IL-17, IL-6 and IL-22 were determined as described in Materials and Methods. Samples of sham and burn mice were collected 3 hrs after burn or sham procedure. Data are mean ± SE; n = 6 mice/group. No significant differences were found on those tissues in the burn group compared to the sham animals. n/d = not detectable

### Burn induces heart injury at 3 hours after burn

**Figure 3:**
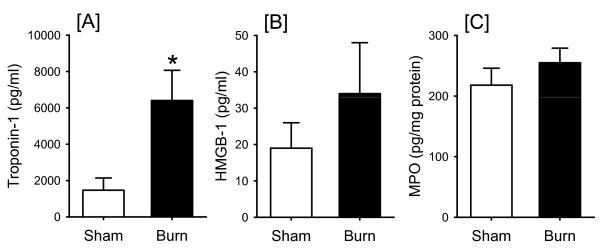
***Cardiac MPO and plasma Troponin and HMGB-1 levels*: At 3 hrs after burn and sham, plasma Troponin (A) and plasma HMGB-1 (B) and cardiac MPO (C) levels were assessed as described in the Materials and Methods**. Data are mean ± SE for 5-6 mice/group. *P < 0.05 as compared to sham.

We found that burn was associated with increased Troponin-I levels at 3 hours after injury (Fig. 3A), indicating cardiomyocyte damage. At 24 hours after the burn, the Troponin-I levels began to normalize and did not differ from those of the uninjured animal (data not shown). Plasma levels of the early inflammatory marker HMGB-1 were not elevated at 3 hrs (Fig 3B). To elucidate the role of neutrophils in the early cardiac injury after burn, we analyzed heart tissue for MPO levels. As shown in Fig. 3C., MPO levels were unchanged in the burn group as compared to the sham animals at 3 hours after burn. Histologic analysis indicated no major changes in cardiac morphology at 3 hrs after burn (data not shown.)

### Burn induced cardiac cytokine response is Th-17 specific

To determine whether the cytokine response in the heart after burn was also related to changes in the Th1 and Th2 we analyzed heart tissue for IFN-γ or IL-10 as specific markers of the Th1 and Th2 response respectively. IFN-γ was not detectable irrespectively of the group and IL-10 levels were similar in sham and burned animals at 3 hrs after injury. (Table 2)

**Table 2:** Cardiac levels of IFN-γ and IL-10

	Sham	Burn
IL-10	87 ± 25	133 ± 28
IFN-γ	n/d	n/d

Cardiac levels of IL-10 and IFN-γ were determined 3 hrs after burn or sham procedure as described in the Materials and Methods. Data are mean ± SE; n = 6 mice/group. IFN-γ was not detectable irrespectively of the group and IL-10 levels were similar in sham and burn mice. n/d = not detectable.

## Discussion

The inflammatory cascade elicited by burn activates a broad immunoinflammatory response known as the Systemic Inflammatory Response Syndrome (SIRS). This response involves a wide range of cells (e.g, macrophages, T-cells, neutrophils) and tissues (e.g., skin, spleen, liver, cardiac, lung). Ultimately, SIRS increases the potential for the development of Multiple Organ Dysfunction Syndrome (MODS). Since the initial studies of Blalock in the 1930's about cardiovascular impairment after burn, many models have been proposed to explain its pathophysiology including fluid shift, changes in the microcirculation, coagulopathy and peripheral vasoconstriction, however, the most recent research studies have been focused on the association of certain factors with the cardiac dysfunction after burns, including complement, cellular apoptosis and cytokines as the purpose of the current study [2,11-14]. The pathophysiologic response in the cardiovascular system includes myocardial contractile dysfunction and increased vascular permeability. Several studies have shown that cardiac depression associated to burn injuries occurs independently of the plasma loss, develops early after the injury and early large volume resuscitation does not affect morbidity or mortality, suggesting an intrinsic tissue damage as a cause of its failure [2,15,16]. Studies have confirmed the cardiovascular function impairment is in part a consequence of increased generation of potentially harmful mediators such as pro-inflammatory cytokines in models of myocardial ischemia, burn and sepsis [17,18]. Studies done by Barber et al, have pointed to cardiomyocytes as the source of those cytokines [3]. This study by Barber et al. also showed a correlation of the burn size and the cytokine response and the degree of left ventricular dysfunction to a nadir of 40% TBSA. Maass et al. showed a synergistic effect of IL-1β, Il-6 and TNF-α exacerbating cardiac contraction and relaxation deficits produced by any one inflammatory cytokine [17].

Studies by Finnerty et al. have recently shown that circulating IL-17 levels are increased early after burn in pediatric patients, as well in a mouse burn model [19,20]. These initial findings are suggestive that IL-17 plays an important role in the inflammatory response after burn. In that regard, results from the present study demonstrate that the early cardiac inflammatory response after burn involves IL-17 and other Th-17 cytokines (IL-6, IL-22). This cardiac Th-17 response correlated with the development of cardiac injury, as reflected in elevated plasma levels of Troponin-I, a sensitive and specific marker of myocardial injury after burn, correlating with cardiac contraction and relaxation deficits [21]. Huang et al. also showed a significant increase in serum levels of Troponin-I at 3 hours after burn [15]. Somewhat surprisingly elevated levels of cardiac IL-17 were observed in sham animals at 3 and 6 hrs. This apparently aberrant response may be related to volume loading due to the administration of 1 ml IP of Ringer's lactate to the sham mice. This concept is supported by the observation that by 1 day once fluid levels have normalized IL-17 levels are barely detectable in the sham mice. In our study, the Th-17 response at 3 hr was organ specific as other tissues (lung, liver and small intestine) did not show a significant change in the Th-17 response. This does not preclude the possibility that other tissues express a Th-17 response later after injury. The recruitment of immune cells, particularly neutrophils, to a site of injury is the initial step in most inflammatory processes. Activated neutrophils release proteases and reactive oxygen intermediates, which can lead to significant tissue damage [22]. Recent studies by Abdel-Rahman showed that limiting neutrophils activity after cardiopulmonary bypass, improves perioperative hemodynamics and cardiac function, demonstrating its detrimental effects upon activation [23]. We have shown that the cardiac Th-17 response at 3 hr. post-burn is not associated with neutrophils infiltration as evidenced with unchanged levels of cardiac tissue MPO. A change in circulating levels of the HMGB-1 was also not evident in the current study. This may be in part related to the early time after injury that was investigated or the severity of the burn.

Evidence points to IL-17 as the major effector molecule produced by Th-17 cells. IL-17 stimulates several cell types, such as endothelial cells, epithelial cells and macrophages to produce multiple pro-inflammatory mediators, including IL-6 and IL-23 [24]. It also stimulates the mobilization and de novo generation of neutrophils by granulocyte-colony stimulating factor (G-CSF), thereby bridging a gap between innate and adaptive immunity and might constitute an early defense mechanism against severe sepsis [25]. Th-17-driven effector functions may also be different in different tissues [7]. We found that cardiac Th cytokine responses after burn were specific for Th-17, as cardiac levels of Th-1 (IFN-γ) and Th-2 (IL-10) cytokines were not significantly affected by burn. IL-17 is thought of as a T-cell derived cytokine, however, limited studies suggest to non T-cell cell types can also produce this cytokine. Recent study by Lapara NJ 3rd et al. showed that macrophages are the key source of inflammatory mediators in Leishmania infection [26]. Studies have also shown increased IL-17-expressing macrophages in active inflammatory bowel disease, high expression of IL-17 in macrophages-mediated inflammatory response in breast cancer as a promoter of invasiveness, and increased production in astrocytes and oligodendrocytes in patients with active multiple sclerosis [27-29]. Thus, the concept that cardiomyocytes can produce IL-17 directly is plausible. Future studies will need to examine the ability of isolated cardiomyocytes to produce IL-17 in vitro to validate this concept.

## Conclusion

Interventions in the complex inflammatory cascade following burn might improve morbidity and mortality in this patient population. These initial findings suggest that IL-17 may provide a unique target of therapeutic intervention to reduce cardiac dysfunction after burn.

## Abbreviations

IL: interleukin; TBSA: total body surface area; TGF-β: Transforming growth factor-Beta; IFN-γ: Interferon gamma; TNF-α: tumor necrosis factor alpha; HMGB-1: High-mobility group box-1; MPO: myleoperoxidase; SIRS: Systemic Inflammatory Response syndrome; MODS: Multiple Organ Dysfunction Syndrome.

## Competing interests

The authors declare that they have no competing interests.

## Authors' contributions

JS and QZ were responsible for the animal experiments and ELISAs. MR was responsible for ELISA analysis, data analysis and scientific interpretation. RO was responsible for the data analysis, scientific interpretation and drafted the manuscript. MS was responsible for scientific conception, design and helped to draft the manuscript. All authors read and approved the final manuscript.
